# Experience of living near a highway in Nepal: Community perceptions of road dangers in Makwanpur district

**DOI:** 10.1016/j.jth.2022.101337

**Published:** 2022-03

**Authors:** Elisha Joshi, Preeti Gautam, Anish Khadka, Paul Pilkington, John Parkin, Sunil Kumar Joshi, Julie Mytton

**Affiliations:** a Nepal Injury Research Centre, Kathmandu Medical College Public Limited, Kathmandu, Nepal; b Centre for Public Health and Wellbeing, University of the West of England, Bristol, UK; c Centre for Transport and Society, University of the West of England, Bristol, UK

**Keywords:** Community perception, Road safety, Highway, Nepal

## Abstract

**Introduction:**

Road traffic injuries are a major but neglected global challenge. There are high and rising rates of road traffic injuries in Nepal. Most of the studies reporting these injuries in Nepal have used quantitative methods to describe the injury burden. Little qualitative research has been conducted to describe the contexts and social processes surrounding crashes, or public perceptions of risks and potential solutions. The aim of this study was to explore the perceptions of road dangers from communities living alongside a major highway in Nepal.

**Methods:**

In this qualitative study we recruited members of neighbourhood development committees and a mother’s group to take part in focus groups exploring their views. Data were audio-recorded, transcribed, translated and analysed thematically.

**Results:**

Four focus groups were conducted involving 34 participants aged 24-65. Our study findings highlight the challenges faced by people living near a major highway and their fear of getting injured on the road. Five themes that emerged were: risky behaviours of road users, infrastructure for safer behaviour, poor condition and maintenance of roads and vehicles, limited adherence and enforcement of traffic laws, and the need for road safety awareness programmes.

**Conclusion:**

The community groups expressed multiple concerns regarding the safety of members of their communities and lived-in fear of death and injury on the road where they lived. There is an urgent need for government agencies to understand these concerns and to take action in relating to infrastructure provision, regulation and behavioural change programmes.

## Highlights

• Communities living near highways in Nepal fear traffic and the risk of injury or death.
• Community groups perceive themselves to have a role in safety education for increasing awareness to bring behaviour change.
• Residents expressed the urgent need of government agencies to listen to their concerns and take action.

## 1 Introduction

Nearly 3700 people die on the world's roads every day in road traffic collisions (RTCs), more than 1·35 million lives per year, and 50 million people sustain road traffic injuries (World Health Organization, 2018). Ninety percent of these fatalities occur in low-and middle-income countries (LMICs), and the South-East Asia region has the second-highest incidence of road traffic injury deaths in the world i.e., 20.7 deaths per 100,000 population (World Health Organization, 2018). There are significant economic impacts of these road crash fatalities and injuries for LMICs; costing them 1.7 trillion dollars and up to 6.5 percent of GDP every year (The World Bank, 2017). Achieving the Global Goal of reducing road traffic deaths by at least 50% from 2020 to 2030 (Third Global Ministerial Conference on Road Safety, 2020) is a major hurdle.

Nepal has challenging road conditions in urban and rural settings due to a lack of adequate road safety infrastructures, difficult topography, and engineering challenges (Pant et al., 2020). Most of the studies reporting RTIs in Nepal explore quantitative data (Joshi, 2009; Karkee and Lee, 2016; Pant et al., 2020; Thapa et al., 2020). The rate of road traffic crashes in Nepal is estimated to be 100 times greater than that in Japan and 10 times that in India (Adhikari, 2016). Global Burden of Diseases (GBD) estimates for Nepal, suggest that 6788 road traffic deaths occurred in 2017, of which 53% were pedestrians, 19% motorcyclists, and 20% of people were in motor vehicles (Global Burden of Disease, 2017). Between 1990 and 2017, pedestrian road injuries were the predominant type of transport injury leading to disability-adjusted life years (DALYs) in both sexes in Nepal (Pant et al., 2020). A study analysing five-years (2012/13–2016/17) of police traffic crash records showed that 73,540 crashes occurred throughout the country resulting in 9808 deaths and 59,501 injuries (Katuwal et al., 2018).

Qualitative studies are essential in traffic injury prevention research by describing the contexts and social processes surrounding crashes (Roberts, 1997; Rothe, 2000), however, such qualitative RTI evidence from Nepal is limited. Participants in a study of 25 semi-structured interviews with stakeholders in Nepal found that most perceived cause of RTIs was largely due to violation of traffic rules by both drivers and other road users, and that there was a need for a heavier system of fines against traffic rule violations to be introduced and that traffic safety awareness programmes to be provided to all road users (Katuwal et al., 2018). A qualitative study using photo-elicitation methods to engage children in a discussion about road safety found that many reported their journey to school, along the East-West Highway to be dangerous highlighting multiple risks including speeding, inappropriate parking, and overloaded vehicles (Gautam et al., 2021). Understanding peoples’ perceptions of safety is important in road design, since their knowledge and understanding of travel attitudes and behaviours would help to recognize potential high-risk circumstances that may not be revealed in official collisions or injury record data (Schneider et al., 2004).

In light of the very limited evidence of community perceptions of road safety in Nepal, this study was designed to capture the views of the general public regarding their beliefs about road traffic crash risks. Such views are important since local residents are more likely to be aware of the nature, cause, and possible solutions of traffic issues (Roberts et al., 1995). To our knowledge, this is the first study exploring the perspective of the general public on road traffic crash risks, and therefore contributes new knowledge for the Nepal context. Therefore, the overall aim of this research is to explore public perceptions regarding road traffic injuries, and factors facilitating and hindering the reduction of road crash risks.

## 2 Methods

### 2.1 Study design

This is a qualitative study using focus groups (FGs) to enable an understanding of community views on the risks of living next to a busy highway in Nepal. This method allowed the exploration of participants’ direct experiences in the socio-spatial context in which they live (Krueger, 1994), and enabled the collection of views, opinions, and perspectives among a diverse range of participants. FGs generate data through interaction with research participants; the discussions highlight the cultural values and enable inclusion of participants who may not be able to read or write (Brown et al., 2006).

### 2.2 Setting

**Fig. 1:**
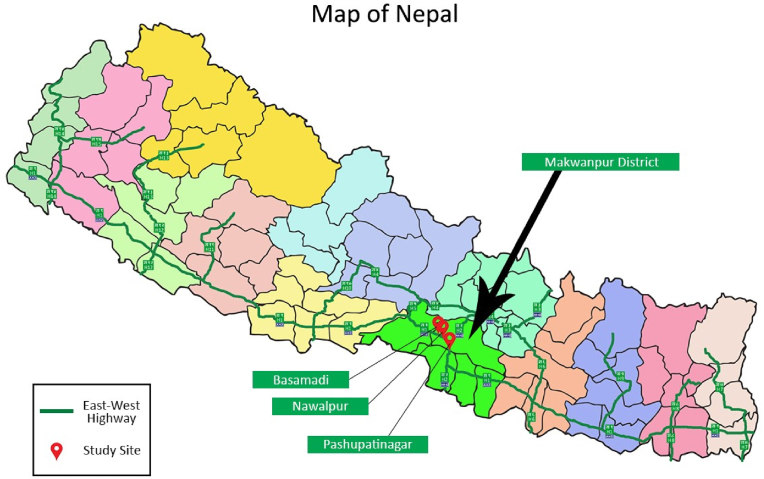
Map of Nepal showing the East- West highway and study sites (Source: https://bit.ly/3xP0Zrw).

This study was embedded in a programme of road safety research conducted in Makwanpur district, Nepal. The study was undertaken near Hetauda, a Sub-metropolitan city in Makwanpur District of Bagmati Province (See Fig. 1). The district has a population of 420,000, with 45% being under the age of 18years (Central Bureau of Statistics, 2012). Having a mixed geographical terrain (i.e., hills, mountains, and plains), it reflects a range of socioeconomic settings found in Nepal (Osrin et al., 2003).

The district, being traversed by two prominent highways (the East-West highway and the Tribhuwan highway connecting south to the Indian border) is busy with commercial traffic and prone to road traffic crashes. The programme of road safety research focussed on the East-West highway near the city of Hetuada, as it serves several urbanized and rural areas and is one of the major trade routes in Nepal (Pokharel and Acharya, 2015). Three study locations (Pashupatinagar (south of Hetauda); and Nawalpur and Basamadi (both west of Hetauda) (as shown in Fig. 1)) were included in the wider programme of road safety research based on an analysis of District traffic police records for the year 1^st^ April 2017 to 31^st^ March 2018 along with site visits and community engagement activities. These sites have high daily traffic volumes: Pashupatinagar- 18,707, Nawalpur: 14,056 and Basamadi- 11,125 vehicles/day respectively.

### 2.3 Participants

The research team visited the local government administrative offices and spoke to local shopkeepers at all three locations to identify existing local community groups. The shopkeepers identified four established community groups: three Neighbourhood Development Committees (NDCs) and one Mother's Group. Neighbourhood Development Committees are established throughout the country and draw membership from around 80% of households in a locality. (Chitrakar, 2016). NDCs are involved in a wide range of development activities including the provision of drinking water, management of waste and sewage, supporting infrastructure development by collecting funds from membership, and planning community improvement activities (Ninglekhu and Rankin, 2008). Mother's groups consist of women engaged in various local social and health activities within their locality (Khatri et al., 2017).Being the main grass-root community groups working in the study sites for community welfare, they were appropriate groups to voice road safety concerns for the communities they represented. Each NDCs we invited had a boundary along the highway at each study site, and, the mother's group was active in community safety, having already established night patrols to support people using drugs or at risk of domestic violence.

### 2.4 Data collection

We collected data from August to September 2019. Before each focus group, a written participant information sheet in Nepali containing a description of the study was provided to all the participants by researchers. Written informed consent was obtained from all the participants, and no one declined to take part. All the FGs were conducted at a time and venue negotiated with the participants, such as at a local school or ward office. A topic guide (See Annex) was developed iteratively by the research team to facilitate discussion on the perception of road dangers while living near a highway, potential preventive measures, and barriers and enabling factors for reducing road traffic crashes locally. Prompts were used to probe and obtain individual expressions and perspectives. The topic guide was initially prepared in English, the content was discussed with other co-authors and then translated into Nepali. The research team led the group discussion with a note-taker recording the group discussion. Additional care was taken to minimize the silencing of individual voices. All the focus groups were conducted in Nepali and audio recorded with participants’ consent.

### 2.5 Data analysis

The FGs were transcribed verbatim in Nepali by two experienced transcribers and then translated into English. The first and second authors (who are both fluent in Nepali and English) read the verbatim transcriptions and compared them to the translated text to ensure accuracy of translation. The researchers de-identified the transcripts by allocating unique identification codes to each group and followed the six-step approach of thematic analysis described by Braun and Clarke (Clarke and Braun, 2013). The translated transcripts were imported into NVivo version 12.0 software to aid the coding process. Two of the authors (EJ and PG) coded the same transcript independently allowing comparison of the identified codes, and a coding framework was then agreed and applied consistently to the remaining transcripts. EJ and PG developed the themes by merging codes describing similar concepts, and the themes were finalized through discussion with AK and JM. We used a number of strategies to reinforce the rigor of our study. These included: trained researchers facilitated the data collection; ensured all participants in the focus group had an opportunity to contribute so that more confident participants did not dominate others; transcripts were anonymised and dual data coding was done; cross-checking of full transcripts was carried out against audio files for quality and completeness; adequate probing and cross-checking was done during each FGs to reduce social desirability bias.

### 2.6 Ethical consideration

Ethical approval for this study was obtained from the Institutional Review Committee of Kathmandu Medical College (KMC) in Nepal and the Health and Applied Sciences Faculty Research Ethics Committee at the University of the West of England (UWE), Bristol, UK. A permission letter was obtained from the Hetauda Sub-metropolitan office, Makwanpur to conduct the study.

## 3 Results

A total of 34 participants were involved in this study. Table 1 presents the sociodemographic characteristics of the participants. Most participants were female (82%), reflecting our inclusion of a Mother's Group, and a higher proportion of women in the NDCs were recruited. Participant's ages ranged between 25 and 65 years and they had varying levels of education (from illiterate to university degree). Thematic analysis of the transcript data led to the development of five overarching themes: (3.1) Risky behaviours of road users, (3.2) Infrastructure for safer behaviour, (3.3) Poor condition and maintenance of roads and vehicles, (3.4) Limited adherence and enforcement of traffic laws (3.5) Need for road safety awareness programmes.

**Table 1:** Profile of respondents participating in the focus groups.

Area		Basamadi NDC	Nawalpur NDC	Pashupatinagar		Total
				Mother's Group	NDC	
Number of participants in focus group		9	10	8	7	34
**Sex**	**Male**	3	1	0	2	6
	**Female**	6	9	8	5	28
**Age**	**25–45**	5	6	2	1	14
	**46–65**	4	4	6	6	20
**Education**	**Illiterate**	1	1	3	0	5
	**Literate**	2	2	0	3	7
	**Primary**	0	1	4	0	5
	**Secondary**	3	2	1	2	8
	**Higher secondary**	2	1	0	2	5
	**University**	1	3	0	0	4

NDC=Neighbourhood Development Committee.

### 3.1 Risky behaviours of road users

Participants reported various behaviours of road users which didn't comply with safety norms neither for themselves nor for those around them, thus increasing the risk of being injured. Driver behaviour gave the greatest concern, but examples of risky behaviour of all road users were given including pedestrians and motorcyclists.

Many participants identified speeding, dangerous overtaking, overloading of vehicles (passenger and freight), driving under the influence of alcohol or drugs and sleep deprivation among drivers as being common causes of RTCs. Participants explained how they did not feel safe while riding the public buses since driven by motive to earn more money the drivers try to get to each bus stop quickly and pick up as many passengers as they can, disregarding the safety of their passengers. There was widespread reporting of the perilous behaviour exhibited by commercial vehicle drivers. One possible explanation given was as the drivers were being paid for each trip they make; they engaged in risky behaviours such as speeding, overtaking and driving for many hours without a rest in order to make more trips and earn more money. Participants believed that such behaviours not being monitored and driving hours related laws not being enforced were the reasons why such risky road behaviours among commercial drivers persisted.

*Nowadays what the public has started to say is ‘the killer is here; the killer is here’. I was scared once. When they say the killer is here you will be scared! They were indicating the tipper … A person will be smashed dead and the driver will still be relaxing in his seat.* [Female, 56 years, FGD2]

*The tripper [truck] drivers are the ones who frequently cause accidents as they overtake and hit the pedestrians walking at the side of the road …* [Female, 36 years, FGD3]

Respondents believed that despite laws preventing drunk driving, drivers tend to indulge in drinks and substance abuse and avoid detection by the police. They gave many examples of road traffic crashes resulting from the driver being under the influence of alcohol or drugs. Respondents highlighted the emerging trend of marijuana consumption that is slowly replacing alcohol since it is not detected at police check points. Participants suggested that while drunk driving checks needed to continue, the police needed a robust mechanism to identify and control driving under the influence of drugs.

*They take marijuana. They come drinking alcohol. The vehicles coming from this way do night stay near there. They come near there taking alcohol because of less possibility of [police] checking in the evening.* [Female, 34 years, FGD1]

Along with the drivers, some participants recognized that people living alongside the road may contribute to the risk of crashes as they explained that local people lacked patience when crossing the road. They described instances where pedestrians had not used nearby zebra crossings but rushed across the road at any convenient location.

*… Even if there are zebra crossings, we don't reach there to cross roads.* [ Male, 36 years, FGD2]

The participants also described people who quite often leave their cattle on the road, hence increasing the risk of RTCs. Participants showed particular concern over adolescents’ behaviour on the road and how this made them vulnerable to road traffic injuries. One of the respondents described how copying others and showing off led to speeding among adolescents who are known to take risks at this stage of their development.

*I didn't allow my own son to ride a bike when he had a license because these youngsters, they just know how to twist the accelerator and make noise. They don't think what may happen ahead.* [Female, 47years, FGD2]

### 3.2 Infrastructure for safer behaviour

A lack of appropriate infrastructure was cited as a barrier to promoting safe behaviours. Respondents identified examples of inadequate infrastructure, including a lack of designated bus stops, places to park, pedestrian footways, pedestrian crossings, bridges to separate pedestrians from motor traffic, and lane separation markings on the carriageway.

*The road should be extended for pedestrians. Two sides of the road should be allocated for pedestrians. There should be separate lanes for small vehicles and heavy vehicles. There should be separate lane for pedestrians.* [Female, 36 years, FGD1]

Participants perceived that, in the absence of designated bus stops, buses picked-up and dropped-off passengers at any location, increasing the risk for all road users, especially if the bus stops unexpectedly, or at inappropriate locations. The absence of designated places to park has resulted in randomly distributed roadside parking. The participants described how a vehicle could hit them when they were forced to walk onto the road to get around a parked vehicle blocking the way for pedestrians. Most participants complained about narrow roadsides, often with vehicles parked on them, meaning they have no other choice other than to walk in the carriageway. Some participants recognized that pedestrians may place themselves at risk of injury or may contribute to the risk of crashes, for example by walking within the carriageway when they do not need to.

*They [vehicles] should not stop everywhere. If they do that then there is a chance of an accident. They should make a rule indicating the place where to stop and where not to.* [Female, 57 years, FGD1]

Many participants perceived that the road had become more chaotic over time, as the number of different types of road users had increased and there was an increasing number of different types of vehicles. They suggested that, in the absence of separate lanes for buses, commercial vehicles, motorcyclists, cyclists and for the pedestrians, road users were unable to travel safely.

*Everything is on the same road. Cycle, pedestrian, small vehicle, large trucks (counting on his fingers), everything is on the same road. And the situation of the road is such. Lots of vehicles have entered the city, small and big. The whole road is chaotic.* [Male, 55 years, FGD2]

Many participants commented on the difficulty faced when crossing the road in the absence of pedestrian crossings. Even where pedestrian crossings exist, the speed of approaching vehicles made the participants scared to cross the road. Some participants suggested that having an overhead footbridge would be preferable, though others argued that even if a bridge or pedestrian crossings were available, there was no guarantee that people would use them.

*There are no zebra crossings because this is a highway. So, we face a lot of difficulties to cross the road in the absence of zebra crossing, we don't know from where to cross the road. If there was a zebra crossing, it would be easier for general people like us.* [Female, 47 years, FGD2]

### 3.3 Poor condition and maintenance of roads and vehicles

In conjunction with the earlier mentioned factors, poor road construction and vehicular maintenance were cited as issues causing RTCs. Participants explained that the East-West highway was nearly half a century old and didn't receive adequate repair and maintenance it needed from the local authorities due to budget constraints and a lack of concerted coordinated efforts to improve road safety. They described the lack of any long-term plan to cope with increasing volumes of traffic suggested that the authorities were negligent towards road safety.

*Mahendra highway was built 45–50 years ago with the investment of World Bank. Both sides were cleared to make the road. The government did not move ahead with this planning. When the government did not move ahead with a plan, the roads just formed wherever there was route. The same track got continued. There was no improvement made in it.* [Male, 55 years, FGD2]

*There is no effort of government for constructing pathway for pedestrian to walk in. There is no place. There is no footpath for people, no path for cycle. There is one road for the cycle, people and all.* [Female, 50 years, FGD1]

However, it was recognized by some participants that wider roads may not necessarily mean safer conditions for pedestrians. One participant remarked that having wider roads without appropriate safety infrastructure is not enough.

*Wider road alone does not reduce accident. Student in Bhaktapur and Kathmandu Road mostly get into accident. The issue of accident is not resolved … If there is wide road, the speed of vehicle also increases like father said, there should be an indication for speed limit for different parts of road* [Male 33, FGD 2]

Some participants described how there was no integrated planning with regards to the locations of electricity poles, drainage, and drinking water pipes. Several participants complained that potholes on the road increased the risk of RTIs as they for a long period of time before being repaired. They also observed that road maintenance was not undertaken promptly.

*There are potholes in our road and accidents happen there. The road is like drainage and we are afraid that auto will fall in that steep part.* [Female, 50 years, FGD1]

Participants recognized how vehicle factors could also influence the risk of road traffic crashes. Most of the participants believed that vehicle maintenance was primarily the drivers’ responsibility. One participant described how some drivers simply continue driving until the vehicle breaks down. If maintenance was done regularly, it may help prevent RTCs.

*While driving, drivers should check whether their vehicles are in good condition or not. Driver needs to know how far the vehicle may go. They should consult with maintenance if required.* [Female, 45 years, FGD1]

### 3.4 Limited adherence and enforcement of traffic laws

Many participants described observing the breaching of road rules by drivers, despite them knowing the traffic rules and regulations. Most participants identified poor enforcement of traffic rules as a reason for increased road dangers There was a general agreement that drivers did not fear prosecution as there was little monitoring of traffic rules and weak penalties. Hence, participants suggested that rigorous law enforcement of the rules and heavy fines against violations would help improve drivers’ attitudes towards road safety. One participant felt that not only the drivers but pedestrians who crossed the road away from designated crossing points should also be fined.

*Traffic rules should be followed by drivers. Drivers know everything but they are careless. If heavy fines are allocated for the drivers for violating the traffic rules, then they might be scared to break the traffic rules. They should be fined heavily for over speeding and for drinking while driving.* [Female, 27 years, FGD3]

Participants expressed their disapproval regarding prevailing corruption. For example, they reported some drivers receiving their licence without taking the exams by bribing transport officials, and that this resulted in poor driving skills and an unsafe road environment.

*These days more licenses are being issued by providing bribe than by passing the test. The beginners go for driving lessons but they buy their license with money rather than by passing the trial.* [Female, 36 years, FGD3]

### 3.5 Need for road safety awareness programmes

Most of the participants felt that education to raise awareness was key to road safety for all road users (children, drivers, and pedestrians). Awareness activities, such as road-shows and documentaries, could be organized in schools and the community. Participants from the Neighbourhood Development Committee in Basamadi felt that the NDC itself should conduct awareness-raising programmes but that a lack of financial support was an important factor hindering them from doing so. However, respecting and heeding the advice of only external experts over the local committee members was a concern felt by some participants.

*To do any programme it is connected to financial resources. We do not have adequate financial resources now. That is why it is not so easy to conduct programmes now.* [Male, 43 years, FGD2]

The study participants reported many road traffic crashes where they called the police officials for first response, and sometimes transported injured patients to hospital. Participants felt that living near the highway remained convenient for them, despite the road risks they face. For example, easy access to transportation led to quicker access to health care.

## 4 Discussion

We used focus groups to explore and describe the understanding of road traffic collisions, their causes and potential preventive measures as perceived by people residing near the East-West highway. According to Nepal Road Standards (2013), highways should be located away from populated areas so as to minimize the disturbance to people from construction activities and noise from moving vehicles and to minimize road traffic crashes. However, many families do live near major road routes and our study findings highlight the challenges faced by such people and their fear of getting injured on the road. Through thematic analysis we identified five themes: Risky behaviours of road users, infrastructure for safer behaviour, poor condition and maintenance of roads and vehicles, limited adherence and enforcement of traffic laws, and the need for road safety awareness programmes.

Road safety issues on the national highways are a major concern in Nepal with crash fatalities are alarmingly high. Over the period from 2014 to 2017, there has been a reported annual average of 1.3 deaths per kilometre across seven sections of highway between Kathmandu to Kakarbhitta, with one of those sections being the East-West highway (The World Bank, 2020). Driving behaviour emerged as a major concern in our study as it significantly affects fatal and non-fatal road crashes (Batool et al., 2018).

With major crashes often involving trucks and buses (The World Bank, 2020), our study participants perceived the greatest harm to come from hazardous behaviours such as speeding and risky manoeuvres by motorcycle drivers and commercial truck drivers. Not giving consideration to safety, the commercial truck drivers tend to be lured to earn more by making more trips in a day leading to rash driving which could be addressed by the local authorities. Bringing change in this trip-based payment system among heavy commercial vehicles might reduce risky driving (speeding, overloading and negligent driving). Conducting special video seminars and training sessions for these drivers and making them aware of the consequences of such driving were suggested as steps towards changing the driving behaviour. Additionally, using cameras at major road junctions can monitor such risky driving among other vehicle drivers as well.

Traffic and highway engineers are continually engaged in working to ensure that the street and highway system is designed and operated such that highway crash rates can be reduced (Garber and Hoel, 2014). However, our study participants recognized that the increase in human and vehicle populations, set against a static highway without regular road safety maintenance and improvement, was a major contributing factor to the road safety crisis. Strong emphasis was laid on building infrastructure such as pedestrian footways, zebra crossings, bus stops, and overhead footbridges to promote safety behaviour. The East-West highway does not meet the criteria set out in the Nepal Road Standards (2013) of providing under/overpass bridges for the movement of people at intervals along the highway (Government of Nepal, M.o.P.I.a.T., Department of Roads, 2013). Similar findings were reported in a study in Bangladesh where appropriately wide shoulders to the carriageway; self-enforcing speed-reducing measures; off-carriageway bus stop facilities; lane delineation devices; and segregated footways contributed to reducing RTCs on national highways (Hoque and Mahmud, 2009). When constructing traffic facilities, it is important to include communities’ input, for example, when deciding the most appropriate location for a zebra crossing, road users views will optimize its usefulness (Sari and Efranto, 2012). In a similar way, local communities should be involved in decisions regarding the installation of road safety infrastructure along the East-West highway. However, any road safety intervention should not be installed without any systematic checks to evaluate its impact on the safe operation of traffic (Batool et al., 2012).

With one exception, study participants had a consensus that increasing the number of lanes within the carriageway would increase safety. A study in Kerala, India found that the fatality rate was three times higher on four-lane divided highways as compared to two-lane sections of the same road carrying similar volumes of traffic (Shaheem and Mohammed, 2006). This suggests that, despite popular local community opinion, without other appropriate design measures to separate the highway from other road users, adding lanes will in fact likely result in more deaths and injuries. In turn, this suggests that an education programme is needed to alert local communities on the risks of asking for, and potentially then receiving, the interventions that unintentionally increase RTI risks.

Local authorities failing to invest in road infrastructure and safety was another concern of our participants. This is there despite there being 21 legal documents and 14 policy documents relating to road traffic injuries or their prevention in Nepal as identified by a recent policy and legislation review study (Pant et al., 2021). The National Transport Policy 2001, Nepal Road Safety Action Plan 2013–2020, and the current 20-year Master Plan for Strategic Road Networks (2002–2022) all include road crash reduction recommendations, but just like most of the policy documents, they include little consideration of the allocation of resources, leadership or robust monitoring and evaluation resulting in all these strategic approaches remaining on paper without implementation (Pant et al., 2021). In low- and middle-income countries, it can be anticipated that with limited resources and lack of political will, issues such as road expansion and building safety infrastructure are not addressed quickly (Kayani et al., 2012). It is already acknowledged that the Nepal government is facing challenges such as poor interagency coordination, inadequate human resources, significant funding constraints, and the absence of periodic evaluations of traffic safety (Thapa, 2013, The World Bank, 2020). This calls upon the authorities at federal, provincial, and local levels to act urgently and responsibly so as to make progress in addressing the five pillars of road safety described in the United Nations Global Plan for the Decade of Action for Road Safety 2011–2020, those that appear in the Nepal National Road Safety Strategy (NRSS) and Road Safety Action Plan (RSAP) (The World Bank, 2020).

Participants reported that even when provided with proper road safety infrastructure, road users may not make appropriate use of many of these facilities. This could be due to a lack of knowledge and awareness as to the correct use of facilities or a negligent attitude towards using such facilities. The low level of awareness of traffic laws and safety behaviours reported among the road users could be mitigated by educational campaigns, as suggested by the participants. Such campaigns could be conducted by existing established community groups. This is consistent with a study from South India where traffic safety education was recommended to increase awareness (Mony et al., 2002). Studies suggest that educational programmes alone are not sufficient, and multifaceted interventions, including public health campaigns, community mobilization, and advocacy are needed to improve safety for all road users (Haghighi et al., 2020). These findings suggest that massive social engagement involving citizens of the city including college and school students in innovative programs could bring awareness and change in behaviour for instance students promoting road safety not only in public but also at home. Further, regular training of drivers (motorcyclists, commercial drivers) to update the driver's knowledge about traffic rules and regulations could also be a catalyst to promote safe driving behaviour.

Although participants were aware of legal provisions for driving including the no drink-drive policy, the drivers tended to show reckless behaviour with not only with drinks but new trend of substance abuse, suggesting that new techniques to detect drugs were needed. Educational campaigns combined with police enforcement appear to be more effective than campaigns without police enforcement (Elvik and Vaa, 2004). Poor legislative enforcement was identified as an important barrier to road safety, in our study. As in a similar study (Tetali et al., 2013), participants suggested that in addition to the promotion of safety educational interventions, adoption and enforcement of appropriate laws, increase in the number of traffic police check posts, and penalties would promote a culture of safety and reduce injury risks.

The strengths of this study include the fact that we were able to identify the already established and functioning local community groups. This helped us gather rich and contextual information, since all the participants in the focus groups knew each other and were not inhibited from expressing their opinions. Our participants did have a range of levels of educational attainment increasing the likelihood that their opinions reflected the spread of views across the wider community. However, due to the practicalities of running a focus group we were forced to limit the number of participants such that we were not able to include all the neighbourhood development committee and mother's group members in our study. Those who did not take part may have had different views from those who participated. The participants who volunteered to take part were predominantly women; a greater proportion of male participants may have generated additional findings. Our findings may not be generalizable outside of the setting in which the study was conducted, although our findings resonate with the findings from other studies, suggesting the potential for replicability.

This paper recommends that a serious and sustained interest from the government along with road safety interventions are needed. Behavioural change in the road users is important especially in terms of discipline and patience to integrate road safety into Nepali culture. A change can be brought by sensitizing people at the community level through media communication, involvement of non-government organizations (NGOs), running nationwide road safety education campaigns and sustained enforcement resulting in encouragement and promotion of responsible road user behaviour. There is a need to conduct observational studies of current road safety infrastructures, the results of which will assist authorities to plan and implement required changes. Our findings also suggest the importance of conducting participatory action research with national, provincial, and local authorities and other key stakeholders to identify the barriers to promoting road safety. Additionally, there is a need to conduct research on identifying what factors promote utilization of safety infrastructures among road users.

## 5 Conclusion

The experiences of community people are often absent in road safety studies. This study highlighted the many concerns of people residing near the East-West highway in Nepal, regarding the safety of members of their communities and how they lived in fear of death and injury on the road. There is an urgent need for the appropriate government agencies to understand such concerns and to make highways safer for all road users. The behaviour of road users is a major cause for concern, with many participants identifying problems of speeding, dangerous overtaking, overloading of vehicles, driving under the influence of alcohol or drugs and sleep deprivation. Better infrastructure that leads to safer behaviours was identified as a priority and this includes designated bus stops; places to park; pedestrian footways, pedestrian crossings, bridges to separate pedestrians from motor traffic; and lane separation markings within the carriageway. Awareness through education, improved infrastructure, strict compliance with and enforcement of traffic rules and regulations along with proper navigation and support from the government are required if Makwanpur's roads are to provide its citizen with safe means of transportation.

## Financial disclosure

This research was funded by the National Institute for Health Research (NIHR) (Project ref 16/137/49) using UK aid from the 10.13039/100013986UK Government to support global health research. The views expressed in this publication are those of the authors and not necessarily those of the NIHR or the UK Department of Health and Social Care.

## CRediT authorship contribution statement

**Elisha Joshi:** Investigation, Formal analysis, Writing – original and final draft. **Preeti Gautam:** Formal analysis, Writing – original draft. **Anish Khadka:** Methodology, Investigation, Writing – original draft, Project administration. **Paul Pilkington:** Conceptualization, Methodology, Writing – review & editing, Supervision. **John Parkin:** Conceptualization, Methodology, Writing – review & editing, Supervision. **Sunil Kumar Joshi:** Conceptualization, Writing – review & editing, Supervision. **Julie Mytton:** Conceptualization, Methodology, Writing – review & editing, Supervision, Funding acquisition.

## Declaration of competing interest

None.

## Data Availability

Data will be made available on request.
