# Supplementary material for: Experience of living near a highway in Nepal: Community perceptions of road dangers in Makwanpur district
Source: J Transp Health. 2022 Mar;24:101337. doi: 10.1016/j.jth.2022.101337 (PMC8924877; doi:10.1016/j.jth.2022.101337)
Supplement: Multimedia component 1 [file mmc1.pdf]

<u>Focus discussion group (FDG) with Local Community</u>

- **Participant's characteristics:**

Age:

Sex:

Professional background:

- **Opening questions:**

1. What do you think are the adverse environmental and social impacts of the East-West highway on your communities? How do you think these issues have changed over the years?

<u>Schedule with 4 topics:</u>

- **Understanding of RTIs and potential risk of the road danger on the road**

1. What is your understanding of what a road traffic crash is? (*Prompt: Can you give an example, please?)*

2. What road traffic crashes have you experienced / witnessed along the East-West highway? Can you describe one instance?

3. Which group of road users do you think are the common victims of road traffic crashes? (Vulnerable road users)

4. Which group of road users do you think are the common perpetrators of road crashes?

- **Causes of RTIs and potential danger on the road**

1. Do you feel it's safe to drive/walk/cross the road along the East-West highway? *(If yes or no, why so? Could you please provide some examples)*

2. What do you perceive to be the main causes of road traffic crashes on the East-West highway in the Makwanpur district? (*Prompt: road infrastructure; driver and pedestrian behavior and attitude on the road*)

3. What do you think drivers and pedestrians violate traffic rules (over-speeding, overloading, illegal road-side parking, jaywalking, walk on the carriageway, etc.) and how do you justify such behaviors?

4. What type of crashes (crashes involving fatality/ major injury/ minor injury/ property damage only) do you think should be reported to the Traffic Police? If you feel certain type of crashes are not worth reporting, why so?

- **Activities undertaken and potential solution to prevent RTIs and reduce danger on the road**

1. Do you have any activities linked to prevention and improvement of road safety? (*Prompt: Awareness programs among road users)*

2. Do you have any suggestions on how road traffic crash could be prevented or reduced? Or how the highway can be made safer?

- *a. What sort of thing might help?*
- *b. Who might do this? (Responsibility)*
- *c. How might this be done?*

3. As a community residing along the highway, facing daily risk of involving in a traffic crash, what do you think you should do to prevent/reduce it?

- **Barriers and facilitators to RTIs prevention**

1. What might be hindering or deterring the road users (drivers, pedestrians, passengers, and institutions) to adopt safer behavior and foster safer road environments to prevent road traffic crash? *(Prompt: lack of awareness, accountability of stakeholders)*

2. What might help or encourage the road users to adopt safer behavior and foster safer road environments to prevent road traffic crash?

- **Closing remarks**

1. Do you have any other comments or anything else that you would like to say?
